# Full-Thickness Retinochoroidal Incision in the Management of Central Retinal Vein Occlusion

**DOI:** 10.1155/2015/853539

**Published:** 2015-02-05

**Authors:** San-Ni Chen, Ya-Chi Huang

**Affiliations:** ^1^Department of Ophthalmology, Changhua Christian Hospital, Changhua, Taiwan; ^2^Department of Medicine, Chung-Shan Medical University, Taichung, Taiwan; ^3^Department of Medicine, Kaohsiung Medical University, Kaohsiung, Taiwan

## Abstract

*Purpose.* To evaluate the clinical outcomes in patients with central retinal vein occlusion (CRVO) treated with full-thickness retinochoroidal incisions and to compare whether there is difference in treatment response in ischemic and nonischemic CRVO. *Methods.* Retrospective study of patients of CRVO receiving full-thickness retinochoroidal incisions in Changhua Christian Hospital. Fluorescein angiography (FA), slit-lamp biomicroscopy, indirect funduscopy, best corrected visual acuity, and central macular thickness (CMT) measured by optical coherence tomography were performed pre- and postoperatively. Patients were divided into an ischemic and nonischemic group according to the findings of FA. Patients were followed up for at least 1 year. *Results.* Twenty-eight eyes (14 ischemic and 14 nonischemic CRVO) were included. Functional retinochoroidal venous anastomosis (RCVA) was achieved in 48 of the 65 retinochoroidal incisions (73.8%). Central macular thickness (CMT) and retinal hemorrhage decreased significantly after the surgery. Significant visual gain was observed postoperatively in the nonischemic group, but not in the ischemic group. Postoperative complications included vitreous hemorrhage (17.8%), neovascular glaucoma (7.1%), and preretinal fibrovasular membrane (10.7%), all of which were in the ischemic group. *Conclusions.* RCVA formation induced by retinochoroidal incisions could improve venous flow, and decrease CMT and retinal hemorrhage. However, only eyes with nonischemic CRVO showed visual improvement.

## 1. Background

Central retinal vein occlusion (CRVO) is one of the major retinal vascular disorders causing severe visual loss. Macular edema and macular ischemia subsequent to CRVO result in significant visual loss [1]. Although grid laser treatment reduces angiographic macular edema [2], it does not improve visual acuity. Recent studies suggest that intravitreal steroid [3] and antivascular endothelial growth factor (anti-VEGF) agents [4, 5] may improve visual acuity and macular edema in CRVO patients with ME. However, intravitreal injections of pharmacologic agents only lessen the macular edema and improve visual acuity temporarily since the venous flow cannot be reestablished, and for this reason, repeated injections are necessary in most cases. Failure to repeat injections may result in unsatisfactory visual results [6].

To create another retinochoroidal venous bypass may be another way to prevent repeated intravitreal injections of anti-VEGF agents. Methods of induction of retinochoroidal venous anastomosis (RCVA) for CRVO with ME include strong laser puncture [7], vitrectomy with radial optic neurotomy [8], vitrectomy and retinochoroidal incisions [9–12], or local infusion of tissue plasminogen [13].

In this retrospective study, we aim to study the therapeutic effect of full-thickness retinochoroidal incisions that induce RCVA in patients with CRVO and macular edema and to determine whether there is difference in the treatment outcome between ischemic and nonischemic CRVO.

## 2. Methods

We conducted a retrospective study of consecutive case series of patients at Changhua Christian Hospital from December 2009 to June 2012 who had CRVO and macular edema managed by vitrectomy, full thickness retinochoroidal incision, and internal limiting membrane peeling. The study adhered to the declaration of Helsinki and was approved by the institutional review board at Changhua Christian Hospital for the review of chart. Inclusion criteria were as follows: an intractable CRVO with ME, which was defined as persistent or relapsed macular edema after at least 2 successive injections of anti-VEGF agents of bevacizumab, onset of disease at least 3 months before surgery and clear ocular media, which would not preclude fundus examination and fluorescein angiography. Exclusion criteria included BCVA equal to or better than 20/40, any history of a severe ocular trauma, glaucoma, vitreous hemorrhage, intraocular surgery other than cataract extraction, and any concurrent pathology of the eye that would affect the surgical result. All patients were followed up at our clinic for at least 1 year.

Clinical examinations including Snellen best corrected visual acuity (BCVA), slit-lamp biomicroscopy, fundus color photography, and fluorescein angiography were reviewed. Preoperative central macular thickness (CMT, 1000 *μ*m central subfield) measured by OCT (Cirrus HD-OCT, Carl Zeiss Meditec) was also recorded. Patients were divided into an ischemic and nonischemic group according to the findings of FA. Patients with a nonperfusion area greater than 10 disc area (DA) were categorized into the ischemic group.

All surgeries were performed by one surgeon (Chen SN). Three-port pars plana vitrectomy, and posterior vitreous detachment induction were performed followed by full-thickness retinochoroidal incisions made at 2 to 3 sites in the second order branch retinal vein at temporal upper and temporal lower aspects or in the first order branch at nasal aspect. A 20 gauge MVR blade (BVI EdgeAhead MVR Knife) was used for bisecting the vein vertically. The knife incised perpendicularly over the vein and all the way down to the choroid, until a firm resistance from the sclera was met. The MVR blade was then pushed forward and backward a little to separate the incised veins and was extended laterally a little. Intraocular pressure was elevated to about 60 mmHg before and 2 minutes after incisions to prevent excessive bleeding. Internal limiting membrane peeling was then grasped with intraocular forceps over the macular area. Sector panretinal photocoagulation was then performed on the drainage area of the distal part of the bisected vein.

All patients were monitored at day 1 and day 8 and then on a monthly basis after surgery for at least 12 months. BCVA, slit lamp biomicroscopy, intraocular pressure measurement, indirect funduscopy, and CMT were performed on each visit. Fluorescein angiography was conducted to evaluate the formation of functional retinochoroidal venous anastomosis (RCVA) 3 months and 6 months and every other 6 months thereafter postoperatively. The definition of functional RCVA is defined according to the previous reports [14], which includes the venous segment of the first order branch vein proximal to anastomosis being thinner, fluorescein angiography with a retrograde venous flow between disc and anastomosis site, and/or a bypass formation at the anastomosis site.

In the nonischemic group, the foveal avascular zone (FAZ) was manually outlined and measured with the Kowa VK-2 image system (Kowa VK-2, Tokyo, Kowa company) by using the fluorescein angiography taken preoperatively and 3 months postoperatively.

Analyses were performed using a commercially available statistical package (SPSS version 11.5 for Windows; SPSS Sciences, Chicago, IL, USA). Snellen BCVA was converted to logMar (logarithm of minimal angle of resolution) BCVA for statistics. Differences in preoperative and postoperative values were evaluated with the Wilcoxon signed rank test and Mann-Whitney *U* test. In all statistical analyses, *P* < 0.05 was considered significant.

## 3. Results

Twenty-eight eyes from 15 male and 13 female patients were included in the study.  Table 1 summarizes the demographic data of all patients. According to fluorescein angiography, 14 were in the ischemic group and 14 in the nonischemic group. The average follow-up time was 21.3 ± 7.8 months.

The mean age of the patients was 62.4 ± 10.7 years (range, 41–85 years). The time from initial visit of CRVO to surgical intervention ranged from 3 to 15 months (mean 5.2 ± 2.8 months). Preoperative Snellen BCVA ranged from hand motion to 20/100 (mean logMar BCVA: 1.45 ± 0.47). All eyes in the ischemic group had initial Snellen BCVA worse than or equal to 20/200 (ranging from hand motion to 20/200, mean logMar VA: 1.68 ± 0.46). In the nonischemic group, all but 2 eyes had initial Snellen BCVA worse than 20/200 (ranging from 20/1000 to 20/100, mean logMar VA: 1.22 ± 0.36). Preoperative CMT ranged from 277 to 802 *μ*m (mean 497.7 ± 143.6 *μ*m). All patients had previous intravitreal injection of bevacizumab (2.8 times of intravitreal injections of bevacizumab on average), and 7 patients had unsuccessful laser retinochoroidal venous anastomosis procedure.

**Figure 1:**
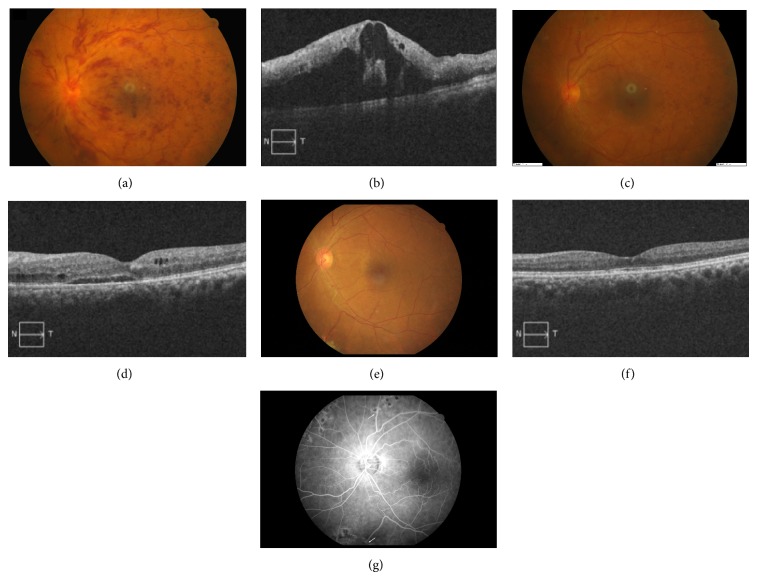
Preoperative fundus picture in patient 11 shows diffuse retinal hemorrhage and macular edema (a). Optical coherence tomography reveals macular thickening with cystic changes (b). Her corrected visual acuity was 20/400. Visible retinochoroidal anastomosis (c), decreased retinal hemorrhage, and decreased macular edema (d) were noted 2 months after surgery. Color fundus photography 1 year after surgery shows a total reabsorption of retinal hemorrhage (e). OCT demonstrates further decreased macular edema (f). Fluorescein angiography shows 2 well-established shunting sites ((g), arrows) and her corrected visual acuity was 20/40.

**Figure 2:**
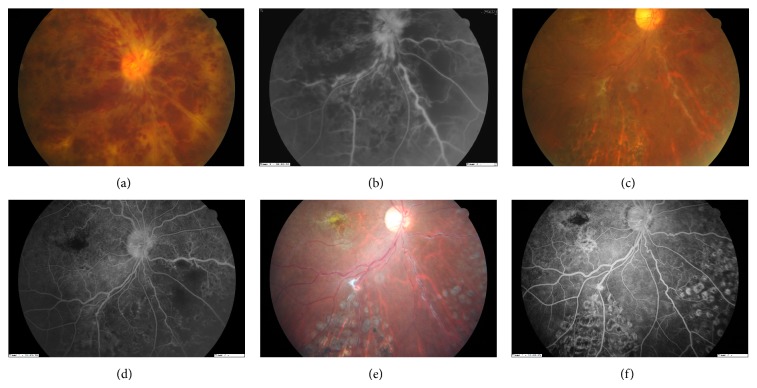
Preoperative color fundus of patient 7 demonstrates diffuse retinal hemorrhage (a). Fluorescein angiography shows a nonperfusion area at the nasal lower retina (b). Color fundus 4 months postoperatively shows a marked decrease in retinal hemorrhage (c); fluorescein angiography demonstrates revascularization toward the originally nonperfused area (d) and retinochoroidal venous anastomosis. Color fundus 4 months postoperatively (e) and fluorescein angiography 18 months later shows almost complete reperfusion of the original ischemic area (f).

In total, 65 retinochoroidal incisions were made in 28 eyes (mean, 2.3 incisions per eye) and 48 functional RCVAs were noted (73.8%). One or more functional RCVAs were observed in all 28 eyes. Visible functioning RCVA was noted from 2 to 4 months postoperatively (Figures 1 and 2). Once developed, the bypassing flow increased throughout the follow-up period.

**Figure 3:**
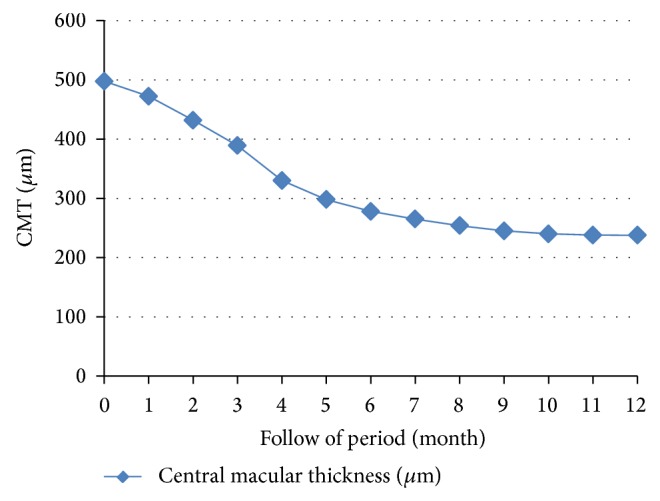
Changes in central macular thickness before and after surgery on a monthly basis.

The mean CMT was 497.7 ± 143.6 *μ*m before the operation and 237.6 ± 86.2 *μ*m at the final visit. The difference in CMT was statistically significant in the whole group and subgroup analyses (Tables 2 and 3, *P* < 0.05, Wilcoxon signed rank test). Reduction in CMT was noted in all eyes and started from 2 to 3 months after surgery and continued to 1 year postoperatively (Figure 3).

Retinal hemorrhage was reduced 1 month postoperatively in all cases, and at 6 months of follow-up, retinal hemorrhage was either largely or completely reabsorbed in all cases (Figures 1 and 2). The reperfusion in the preoperative nonperfusion area was noted in 1 eye (Figure 2). Aggravated nonperfusion was noted in 1 eye. The reperfusion process started to develop about 3 to 6 months postoperatively.

For the visual acuity, the mean logMar BCVA before the operation was 1.45 ± 0.47, which significantly improved to 1.17 ± 0.69 at the final visit (*P* = 0.008, Table 2). For the subgroup analysis, patients in the ischemic group had a mean logMar BCVA of 1.68 ± 0.46 before the operation and 1.64 ± 0.20 at the final visit, which was not statistically significant (*P* = 0.854, Wilcoxon signed rank test, Table 3). Final BCVA improved in 2 eyes, deteriorated in 2 eyes, and stabilized in 10 eyes. None of the eyes had a final BCVA equal to or better than 20/200. In the nonischemic group, the mean logMar BCVA was 1.22 ± 0.36 before the operation and 0.71 ± 0.67 at the final visit, which was significantly different (*P* = 0.008, Wilcoxon signed rank test, Table 3). Nine eyes had final BCVA improve 2 lines or more; the other 5 eyes maintained preoperative vision. Ten eyes had final BCVA greater than 20/200. To further analyze the difference in eyes with stable or improved vision in the nonischemic group, patients with improved BCVA had a younger mean and median age (56.1 y/o and 53.0 y/o) as compared to patients without improvement (71.4 y/o and 70.0 y/o) with statistical significance (*P* = 0.009, Mann-Whitney *U* test, Table 4). Postoperative fluorescein angiography showed a maximal diameter greater in the nonimproved versus improved group (1.57 ± 0.24 mm versus 0.89 ± 0.19 mm, *P* = 0.003, Mann-Whitney *U* test, Table 4). Patients with improved BCVA also had a marginally better mean initial logMar BCVA as compared to the nonimproved group (1.06 ± 0.2 versus 1.50 ± 0.45, *P* = 0.052, Table 4).

Postoperative complications in addition to cataract was noted in 6 eyes, including vitreous hemorrhage in 5 eyes (17.8%), neovascular glaucoma in 2 eyes (7.1%), and preretinal fibrovascular membrane over the incision sites in 3 eyes (10.7%), which were all in the ischemic group. Premacular fibrosis did not develop in any of the 28 eyes. The eyes with vitreous hemorrhage had hemorrhage reabsorbed after IVI bevacizumab and subsequent laser therapy was performed. The eyes with neovascular glaucoma went through IVI bevacizumab and panretinal photocoagulation. One eye needed trabeculectomy to stabilize the intraocular pressure. The eyes with preretinal fibrovascular membrane had the membrane localized without traction on the fovea.

## 4. Discussion

Surgically induced RCVA was first proposed by Peyman et al. [9], who performed slit-like incisions adjacent to the 4 major retinal veins and inserted small pieces of sutures into the incisions in 5 eyes with ischemic CRVO; they reported functional RCVA formation was achieved in 60% of the incisions. Koizumi et al. [10] carried out the complete cutting of the affected retinal vein and small retinal incisions down to the Bruch's membrane on both sides of the punctured vein, with functional RCVA being achieved in 5 of 7 eyes and 3 of the 5 eyes with functional RCVA having visual improvement. Kang et al. [12] reported full-thickness, 1 mm width venous incision extending to the retinal pigment epithelium, Bruch's membrane, and choroid in 9 eyes with nonischemic CRVO. Successful RCVA formation developed in 74% of the incisions and significantly improved central macular edema was noted postoperatively. In our series, a similar rate of functional RCVA formation (73.8%) was achieved, and all eyes developed one or more functional RCVA. Markedly decreased macular edema was noted in every patient.

However, despite all of these indications, visual improvement reached statistical significance only in the nonischemic group. This finding contrasts with previous report of intravitreal injection of anti-VEGF agents, in which eyes with ischemic and nonischemic CRVO both benefited from treatment of anti-VEGF agents [15]. The current study also differs from another report, in which eyes with ischemic CRVO showed significant visual improvement [11]. Recent reports have shown that anti-VEGF agents may reverse retinal nonperfusion and that VEGF promotes retinal nonperfusion in patients with retinal vein occlusion [16]. Since full-thickness retinochoroidal incisions and bisecting retinal veins actually render the distal tributary area of the bisecting veins even more ischemic by completely blocking the venous return before the development of RCVA, the procedures may induce more VEGF release postoperatively, which may further deteriorate the macular nonperfusion status. This may explain why there is no visual improvement in eyes with ischemic CRVO, despite the resolution of macular edema. For the nonischemic group, postoperative foveal perfusion also seemed to play an important role determining the prognosis. Eyes with improvement had statistically smaller mean FAZ postoperatively than that in eyes in the nonimproved group (*P* = 0.043, Mann-Whitney *U* test, Table 4). Eyes with improvement also had on average a reduced FAZ postoperatively in contrast to the nonimproved eyes in which the FAZ enlarged postoperatively (Table 4). The results show that in the nonischemic group, even though the venous flow was reestablished postoperatively, the changes of the avascular zone secondary to the surgical procedures were probably the determining factor of visual improvement. In the nonischemic eyes, preoperative BCVA and younger age were noted as a marginally positive and positive prognostic factor for visual improvement (*P* = 0.052 and 0.009, resp., Mann-Whitney *U* test, Table 4). According to Epstein et al., younger age was a positive prognostic factor for visual improvement in patients treated with anti-VEGF agents, and patients older than 70 years of age fared less from intravitreal injection of bevacizumab for the treatment of CRVO [17]. This may reflect the fact that patients with better preoperative BCVA and younger ages tend to have a better preserved perifoveal capillary network, which would be more resistant to increased hydrostatic pressure brought by CRVO and also more resistant to the deleterious effect of postoperative VEGF-surge on foveal perfusion. Thus, a visual gain could be obtained after reestablishment of the venous return.

In addition to the improvement of venous flow, reperfusion of the nonperfused area is another goal for RCVA. Unfortunately, in our serial follow-up, reperfusion was noted to develop in only 1 eye in the ischemic group (Figure 2). Further, progression of nonperfusion was noted in 1 eye (ischemic group). The persistent or even aggravated nonperfusion in the ischemic group could explain why recurrent vitreous hemorrhage and NVG developed in 5 cases of ischemic CRVO, even though all the eyes had laser photocoagulation performed at the distal tributary area of the bisecting veins. For this reason, PRP should be performed in every case of ischemic CRVO undergoing bypass surgery. The failure of reperfusion in most eyes may also indicate that the resolution of macular edema was largely due to the reduction of venous pressure by the functioning RCVA instead of the reduction of VEGF.

A weakness of this study is the small population, though it is the largest studied group of surgically induced RCVA up to now. In conclusion, surgically induced RCVA may effectively reduce macular edema secondary to CRVO; visual gain is more likely to be achieved in the nonischemic group. Given that recurrent vitreous hemorrhage and NVG are not uncommon complications in ischemic CRVO, only eyes with nonischemic CRVO may benefit from this procedure. Further studies with larger numbers of cases and control groups are warranted to validate the risks and the benefits of this approach.

## Figures and Tables

**Table 1:** Demographic data of patients.

	Perfusion statue	Duration of symptoms (months)	Preoperative Snellen BCVA	Postoperative Snellen BCVA	Preoperative CMT (*μ*m)	Postoperative CMT (*μ*m)	Number of RCVA formation	Complication
1/F/75	I	3	1/20	4/200	277	213	1	None
2/M/58	I	4	HM	4/200	388	93	1	VH + NVG
3/M/60	I	6	4/200	4/200	445	138	2	None
4/F/72	I	10	4/200	4/200	309	182	1	None
5/F/63	N	4	4/20	8/20	502	381	2	None
6/F/74	I	5	4/200	4/200	753	229	1	VH
7/F/45	I	5	4/200	4/200	358	283	2	None
8/M/58	I	4	4/200	1/20	409	343	1	None
9/F/41	I	4	1/20	1/20	328	148	2	None
10/F/63	N	9	4/200	4/200	392	201	2	None
11/F/50	N	4	1/20	10/20	409	230	2	None
12/F/59	N	6	1/20	6/20	574	325	1	None
13/M/73	I	3	2/20	4/200	749	160	3	VH
14/M/66	I	3	2/200	2/200	562	202	1	VH + PFM
15/F/73	N	3	2/20	16/20	414	225	1	None
16/M/62	I	4	4/200	4/200	380	182	2	PFM
17/M/71	I	4	4/200	4/200	772	169	2	VH + NVG
18/M/46	N	3	2/20	6/20	535	444	3	None
19/M/47	N	3	1/20	8/20	802	305	2	None
20/M/85	N	15	4/200	4/200	464	199	2	None
21/M/76	N	5	4/200	4/200	599	355	2	None
22/M/70	N	3	4/200	4/200	527	189	1	None
23/M/62	I	11	4/200	4/200	385	224	2	PFM
24/F/68	I	8	1/20	1/20	476	198	2	None
25/F/53	N	3	2/20	10/20	492	172	2	None
26/M/58	N	3	2/20	20/20	581	168	1	None
27/M/62	N	1	2/20	18/20	626	352	2	None
28/F/57	N	3	4/20	10/20	427	344	2	None

BCVA: best corrected visual acuity, HM: hand motion, CMT: central macular thickness, RCVA: retinochoroidal venous anastomosis, VH: vitreous hemorrhage, NVG: neovascular glaucoma, and PFM: preretinal fibrovascular membrane.

**Table 2:** Comparison of pre- and postoperative central macular thickness and best corrected visual acuity in logMar.

CRVO	Mean	SD	Median	*P* value
Preoperative CMT (*μ*m)	497.7	143.6	470	
Postoperative CMT (*μ*m)	237.6	86.2	207.5	**<0.001**
Preoperative BCVA (logMar)	1.45	0.47	1.50	
Postoperative BCVA (logMar)	1.17	0.69	1.70	**0.008**

CRVO: central retinal vein occlusion, CMT: central macular thickness, BCVA: best corrected visual acuity, and logMar: logarithm of minimal angle of resolution.

*P* value by Wilcoxon signed ranks test.

**Table 3:** Comparison of pre- and postoperative central macular thickness and best corrected visual acuity in logMar in the ischemic and nonischemic subgroups.

CRVO-ischemia (*N* = 14)	Mean	SD	Median	*P* value	CRVO-nonischemia (*N* = 14)	Mean	SD	Median	*P* value
Preoperative CMT (*μ*m)	470.8	170.9	398.5		Preoperative CMT (*μ*m)	524.6	109.7	514.5	
Postoperative CMT (*μ*m)	197.4	62.0	190	**0.001**	Postoperative CMT (*μ*m)	277.9	90.0	267.5	**0.001**
Preoperative BCVA (logMar)	1.68	0.46	1.70		Preoperative BCVA (logMar)	1.22	0.36	1.15	
Postoperative BCVA (logMar)	1.64	0.20	1.70	0.854	Postoperative BCVA (logMar)	0.71	0.67	0.45	**0.008**

CRVO: central retinal vein occlusion, CMT: central macular thickness, BCVA: best corrected visual acuity, and logMar: logarithm of minimal angle of resolution.

*P* value by Wilcoxon signed ranks test.

**Table 4:** Comparison of pre- and postoperative foveal avasculalr zone, pre- and postoperative central macular thickness and age in the improved and non-improved subgroups in the non-ischemic group.

	Improved group (*N* = 9)				Non-improved group (*N* = 5)			*P* value
	Mean	SD	Median		Mean	SD	Median	
Preoperative diameter of FAZ (mm)	1.30	0.17	1.33	Preoperative diameter of FAZ	1.41	0.41	1.21	0.947
Postoperative diameter of FAZ (mm)	0.89	0.19	0.88	Postoperative diameter of FAZ	1.57	0.24	1.47	0.003
Preoperative CMT (*μ*m)	540.0	126.0	535.0	Preoperative CMT (*μ*m)	496.8	76.6	502.0	0.549
Postoperative CMT (*μ*m)	285.0	92.4	305.0	Postoperative CMT (*μ*m)	265.0	94.6	201.0	0.947
Preoperative BCVA (logMar)	1.06	0.20	1.00	Preoperative BCVA (logMar)	1.50	0.45	1.7	0.052
Postoperative BCVA (logMar)	0.27	0.18	0.30	Postoperative BCVA (logMar)	1.50	0.45	1.7	0.002
Age	56.1	8.4	53	Age	71.4	8.4	70	0.009

FAZ: foveal avasculalr zone, CMT: central macular thickness, BCVA: best corrected visual acuity, logMar: logarithm of minimal angle of resolution.

*P*-value by Wilcoxon Signed Ranks Test.
